# Surface charge of the C-terminal helix is crucial for antibacterial activity of endolysin against Gram-negative bacteria

**DOI:** 10.1186/s12929-025-01133-x

**Published:** 2025-03-22

**Authors:** Joonbeom Kim, Su Min Son, Eunbyeol Ahn, Haejoon Park, Sangryeol Ryu

**Affiliations:** 1 Department of Food and Animal Biotechnology, Research Institute for Agriculture and Life Sciences, Seoul National University, Seoul, Republic of Korea; 2 Department of Agricultural Biotechnology, Seoul National University, Seoul, Republic of Korea; 3 Center for Food and Bioconvergence, Seoul National University, Seoul, Republic of Korea; 4 Present Address: COSMAX BTI, R&I Center, Seongnam, Republic of Korea

**Keywords:** Antibiotic-resistance, Gram-negative bacteria, Endolysin, C-terminal amphipathic helix, Positive surface-charge

## Abstract

**Backgrounds:**

Endolysins are promising alternatives to antibiotics because they can lyse bacterial cells rapidly with a low risk of resistance development, however, their effectiveness against Gram-negative bacteria is hindered by the presence of the outer membrane present in Gram-negative bacteria. Several endolysins with amphipathic helices at the C-terminus have been reported to have intrinsic antibacterial activity against Gram-negative bacteria but their action mechanism is not fully elucidated.

**Methods:**

The sequence alignment analysis was assessed with the CLC Main workbench 7, and His-tagged endolysins were purified with affinity chromatography. Site-directed mutagenesis was used to generate mutations in the endolysin to make various endolysin mutants. The muralytic activity of the endolysin against Gram-negative bacteria was analyzed using a turbidity reduction assay and the antibacterial activities of the endolysins were assessed through a viable cell counting assay.

**Results:**

We identified two endolysins, LysTS3 and LysTS6, both of which have similar sequences and structures including the amphipathic helices at their C-terminus. LysTS6 exhibited significantly higher antibacterial activity against Gram-negative bacteria compared to LysTS3 even though both enzymes have similar muralytic activity against the outer membrane-permeabilized Gram-negative bacteria. Systematic truncation and bioinformatic analysis of these two endolysins revealed a major difference in the charge on the surface of their C-terminal helices, suggesting the possibility that the charge on this helix can determine the antibacterial activity of the endolysins against Gram-negative bacteria. We could enhance the activity of LysTS3 against Gram-negative bacteria by replacing Ala_156_ and Glu_160_ with lysine and alanine, respectively, the amino acid residues at the structurally equivalent positions in LysTS6. A similar activity boost was also seen in LysSPN1S and LysJEP4 when the surface charge of the C-terminal amphipathic helix was altered to be more positive through the modification of the surface-exposed amino acid residues.

**Conclusions:**

The antibacterial activity of endolysin against Gram-negative bacteria could be enhanced by adjusting the surface charge on the C-terminal amphipathic helix to more positive, suggesting that the positive surface charge on the C-terminal amphipathic helix of endolysin is crucial for its penetration of outer membrane to reach peptidoglycan layer of Gram-negative bacteria.

**Supplementary Information:**

The online version contains supplementary material available at 10.1186/s12929-025-01133-x.

## Background

The introduction of antibiotics has substantially reduced the incidence of life-threatening bacterial infections. However, the misuse and abuse of antibiotics have led to the emergence of ‘superbugs’ that are resistant to various antibiotics [1]. The World Health Organization (WHO) has reported that the number of deaths caused by these bacteria has exceeded 35,000, indicating that the problem of 'superbugs' has reached a serious level. WHO released a list of priority pathogens for antibiotic resistance, most of which were Gram-negative bacteria. In addition, WHO categorized the severity of bacteria based on risk criteria, noting that all strains falling into the 'critical' category were Gram-negative bacteria as well. These bacteria cause severe infections with high mortality, leading to serious medical problems such as sepsis and even death [2]. Despite efforts to eliminate these pathogens, they are responsible for numerous nosocomial infections, posing a substantial threat to public health [3–8]. Therefore, the development of alternatives to antibiotics for controlling these bacteria still remains as an urgent and critical challenge.

Endolysins are enzymes produced by bacteriophages at the end of their replication cycle. They facilitate the release of progeny phages from the host bacterial cell by specifically degrading bacterial cell walls. When applied exogenously, endolysins can rapidly degrade the cell wall of susceptible bacteria, leading to cell lysis [9–12]. Endolysin exhibits potent antibacterial activity against Gram-positive bacteria in which the peptidoglycan layer is externally exposed [13, 14]. Recent research has also demonstrated the ability of endolysins to destroy Gram-positive bacteria embedded in biofilm matrices, disrupting biofilm structure [15–17]. However, endolysins have limitations in controlling Gram-negative bacteria because the peptidoglycan layer of the Gram-negative bacteria is protected by an outer membrane [18]. The outer membrane comprises phospholipid and lipopolysaccharides (LPS), which prevent the passage of hydrophilic macromolecules to the cell wall [19].

Several endolysins with intrinsic antibacterial activity against Gram-negative bacteria have been reported and these endolysins are found to possess amphipathic helices at the C-terminus that facilitate interactions with lipid components of bacterial outer membrane [20–30]. Certain C-terminal amphipathic helix of endolysin can destabilize the outer membrane of Gram-negative bacteria and allow the endolysin to penetrate the outer membrane [31]. However, the antibacterial activities of these endolysins against Gram-negative bacteria in practical application are generally low [32] that it is necessary to understand the function of the C-terminal amphipathic helix of endolysin further to develop a strategy that can improve its antibacterial activity against Gram-negative bacteria.

In this study, we identified two endolysins, LysTS3 and LysTS6, featuring amphipathic helices at the C-terminus. Both endolysins exhibited similar muralytic activity against membrane-permeabilized *E. coli*. However, LysTS6 showed stronger antibacterial activity and broader spectrum against Gram-negative bacteria compared to LysTS3 without membrane-permeabilization. Our study revealed that modifying the surface-exposed Ala_156_ and Glu_160_ amino acid residues of LysTS3 to lysine and alanine, as in LysTS6, significantly enhanced its antibacterial activity against Gram-negative bacteria. In addition, modification of the surface charge to be more positive on the C-terminal helix could increase the antibacterial activities of LysJEP4 and LysSPN1S against Gram-negative bacteria. These results indicate that the charge on the surface of the C-terminal amphipathic helix is crucial for the antibacterial activities of these endolysins against Gram-negative bacteria and that modifying the surface charge of the C-terminal amphipathic helix is a promising strategy to boost the antibacterial activity of endolysins containing C-terminal amphipathic helices against Gram-negative bacteria.

## Methods

### Bacterial strains and growth conditions

All bacterial strains were cultured in Luria–Bertani (LB; BD Difco, USA) medium with aeration at 37 ºC. The bacterial strains used in this study are listed in Table S1. When necessary, the medium and agar were supplemented with antibiotics or chemical agents at the following concentrations: kanamycin (Km, 50 μg/mL) and isopropyl β-d-thiogalactopyranoside (IPTG, 0.5 mM).

### Molecular cloning

The native endolysin genes were amplified using specific primers through polymerase chain reaction (PCR). Genes of the mutant endolysin were amplified through overlap PCR. In brief, primers containing modified amino acid residues were designed upstream and downstream of the site where the amino acid residues was changed. Then, forward gene fragments and reverse gene fragments were selectively amplified using the designed primers in PCR. The amplified two gene fragments were overlapped without primers for 12 cycles in PCR. Subsequently, the overlapped genes were amplified with the overlap primer sets for 18 cycles. The PCR products of both native and mutant endolysin genes, along with the pET-28α vector (Novagen, Madison, WI), were digested by restriction enzymes BamHI and SalI to include an N-terminal hexa histidine sequence and ligated using the Rapid DNA ligation kit (Roche, Switzerland). The recombinant plasmids were transformed into *E. coli* BL21(DE3) and selected on LB agar containing 50 μg/mL kanamycin. The plasmids used in this study are shown in Table S2.

### Protein purification and in silico analysis

*E. coli* BL21(DE3) harboring the constructed recombinant plasmids were exponentially grown to reach an optical density (OD_600_) of 0.8–1.0 at 37 ºC and induced with 0.5 mM IPTG at 18 ºC for 18 h. The bacterial cells containing over-expressed endolysins were harvested by centrifugation at 10,000*g* for 5 min and resuspended in reaction buffer (20 mM HEPES, 150 mM NaCl, pH 7.4). The resuspension was disrupted by a sonicator (Branson Ultrasonics, USA), followed by centrifugation at 16,000*g* for 30 min. Affinity chromatography using nickel-nitrilotriacetic acid (Ni–NTA) superflow column (Qiagen Gmbh, Germany) was performed to achieve high purity of the extracted endolysins. After the supernatant was co-incubated with Ni–NTA for 1 h at 4 ºC, the resins bound with the endolysins were washed with the reaction buffer containing 20 mM, 40 mM, and 50 mM imidazole sequentially. The endolysins were then eluted using the elution buffer (20 mM HEPES, 150 mM NaCl, pH 7.4, 300 mM imidazole). The expressions of the obtained endolysins were confirmed using sodium dodecyl sulfate–polyacrylamide gel electrophoresis (SDS-PAGE). Finally, the buffer containing purified endolysins was desalted using the PD Miditrap G-25 (GE Healthcare, UK). Protein concentration was determined using the Pierce™ BCA Protein Assay Kit (Thermo Scientific, USA). The sequence alignment analysis between the endolysins was assessed with CLC Main Workbench 7 (Qiagen Gmbh, Germany), and predicted three-dimensional structures of the endolysins were generated by using Alphafold-3.

### Muralytic activity

Exponentially grown *E. coli* ATCC 25922 were resuspended in 0.5 M ethylenediaminetetraacetic acid (EDTA; Avantor, USA) for 30 min. The membrane-permeabilized cells were washed three times and resuspended with buffer (20 mM HEPES, pH 7.4) to an optical density of approximately 0.6 in a 96-well cell culture plate (SPL, Korea). Then, 180 μL of bacterial cells were dispensed in each well, and treated with 20 μL of endolysins at various concentrations (0 to 1000 nM). The decrease in turbidity of each well was measured at 600 nm of wavelength for 2 h at 37 ºC using SpectraMax i3 Plus Microplate Spectrophotometer (Molecular Devices, USA). A standardized calculation method previously described [33] was applied to quantify the muralytic activity of endolysins.

### Antibacterial activity assay

Exponentially grown *E. coli* ATCC 25922 were resuspended in reaction buffer (20 mM HEPES, pH 7.4) and diluted to 10^5^ CFU/mL. Subsequently, bacterial cells were incubated with endolysins at various concentrations (0 to 1 μM) at 37 ºC for the designated incubation time with shaking. Time-kill kinetics were determined by treating 1 μM of endolysins with the cells at 37 ºC for 2 h with shaking. The same aliquots were extracted from each sample at the indicated time points (0, 15, 30, 45, 60, and 120 min). To assess the antibacterial activity of the endolysins against other Gram-negative strains, exponentially grown Gram-negative strains listed in Table S1 were resuspended in reaction buffer and diluted to about 10^5^ CFU/mL. The bacterial suspensions were then treated with 1 μM of each endolysin, followed by the incubation at 37 ºC for 1 h with shaking conditions. The samples were then serially diluted and spotted onto LB agar and incubated overnight at 37 ºC. All assays were performed in triplicate.

### Scanning electron microscopy

Exponentially grown *E. coli* ATCC 25922 were resuspended in reaction buffer (20 mM HEPES, pH 7.4) and adjusted to an OD_600_ of 0.5. The bacterial cells were treated with 1 μM of endolysin LysTS3 and LysTS6 for 15 min and washed 3 times with PBS. The cells were then pre-fixed with Karnovsky’s fixative at 4 ºC overnight. After being washed three times with 0.05 mM sodium cacodylate buffer, the cells were post-fixed using 1% osmium tetroxide at 4 °C for 1 h. The fixed cells were then washed three times with the distilled water and dehydrated with a graded series of ethanol (30, 50, 70, 80, 90, and 100%) and hexamethyldisilazane. After drying and coating, the bacterial cells were examined using a scanning electron microscope (SEM: Carl ZEISS, SIGMA 360, Germany).

### Membrane permeability assay

Disruption of the outer membrane was determined by using the N-phenyl-1-napthylamine (NPN; Sigma-Aldrich, USA) uptake assay as described previously [34]. In brief, exponentially grown *E. coli* ATCC 25922 were washed with PBS and resuspended in the buffer (5 mM HEPES, pH 7.4, 5 mM glucose). The bacterial cells were diluted to reach an OD_600_ of 0.5 and treated with 1 μM of endolysins and NPN (final conc. = 10 μM), followed by incubation at 37 ºC for 30 min. The fluorescence was then measured using a SpectraMax i3 Plus Microplate Spectrophotometer (Molecular Devices, USA) with an excitation wavelength of 350 nm and an emission wavelength of 420 nm.

Disruption of the inner membrane was evaluated by using the SYTOX green Nucleic Acid stain (Thermofishser, USA). Briefly, exponentially grown *E. coli* ATCC 25922 were washed with PBS and resuspended in the buffer (20 mM HEPES, pH 7.4). The bacterial cells were diluted to reach an OD_600_ of 0.5 and were treated with 1 μM of endolysins and SYTOX (final conc. = 1 μM), followed by incubation at 37 ºC for 30 min. The fluorescence was then measured using SpectraMax i3 Plus Microplate Spectrophotometer (Molecular Devices, USA) with an excitation wavelength of 488 nm and emission wavelength of 522 nm. All fluorescence is indicated as relative fluorescence units (RFU).

### Statistical analysis

Statistical analysis was performed by using GraphPad Prism Version 8 (GraphPad Software, USA). Student’s paired t-test and one-way analysis of variance (ANOVA) with Tukey’s multiple-comparison tests were used to compare the results between and among groups, respectively. A P-value of less than 0.05 means a statistically significant difference.

## Results

### Identification and expression of LysTS3 and LysTS6

The genomes of bacteriophages TS3 and TS6 were annotated in the previous study [35] and the identified putative endolysins consisting of 162 and 160 amino acid residues were designated as LysTS3 and LysTS6, respectively. These two endolysins showed high sequence similarity with each other (98% query, 80.5% identity) (Fig. S1A) and had the N-terminal enzymatically active domains homologous to the glycoside hydrolase family 24 (IPR002196) that cleaves the *β*-1,4 linkage between *N*-acetyl-d-glucosamine and *N*-acetylmuramic acid in the peptidoglycan layer. The three-dimensional structures of both endolysins predicted by Alphafold-3 exhibited a high degree of structural similarity (Fig. S1B). The genes of LysTS3 and LysTS6 were amplified using PCR, digested, and ligated into pET-28α vectors containing N-terminal hexa-histidine. The constructed recombinant plasmids were transformed into *E. coli* BL21(DE3) and endolysins were overexpressed. The purified recombinant endolysins showed a single band between 20 and 25 kDa on SDS-PAGE, consistent with their predicted molecular weights of the LysTS3 and LysTS6 (21 kDa and 20.8 kDa, respectively), confirming that the endolysins were successfully overexpressed and purified (Fig. S2).

### Differential antibacterial activity of LysTS3 and LysTS6 against Gram-negative bacteria

**Fig. 1:**
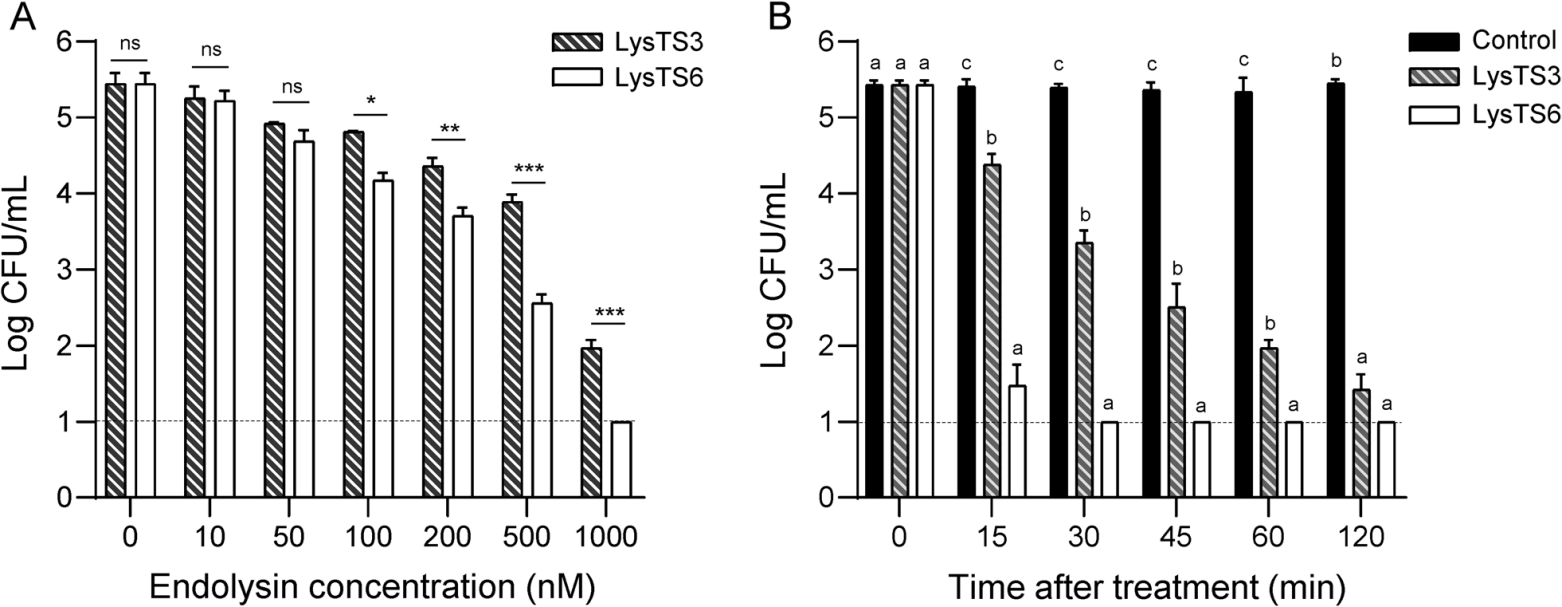
Antibacterial activity of LysTS3 and LysTS6 against *E. coli* without membrane permeabilization. **A** Dose-dependent antibacterial activity of LysTS3 and LysTS6. Exponentially grown *E. coli* were treated with each endolysin at various concentrations, incubated at 37 °C for 1 h, and CFUs were measured. Data represent mean ± standard deviation and the dotted horizontal lines represent the limit of detection. Statistical significance of the difference in the antibacterial activity between LysTS3 and LysTS6 was analyzed by Student’s t-test. *P < 0.05; **P < 0.01; ***P < 0.001; ns, not significant. **B** Time-dependent antibacterial activity of LysTS3 and LysTS6. Exponential phase *E. coli* were incubated with 1 μM of each endolysin at 37 °C and CFUs were measured at designated time points. The endolysin storage buffer served as a negative control. All experiments were performed in triplicate. Data represent mean ± standard deviation and the dotted horizontal lines represent the limit of detection. Statistical significance was analyzed by one-way analysis of variance (ANOVA) with Tukey’s multiple-comparison test among the experimental groups. The different letters indicate statistically significant differences

**Fig. 2:**
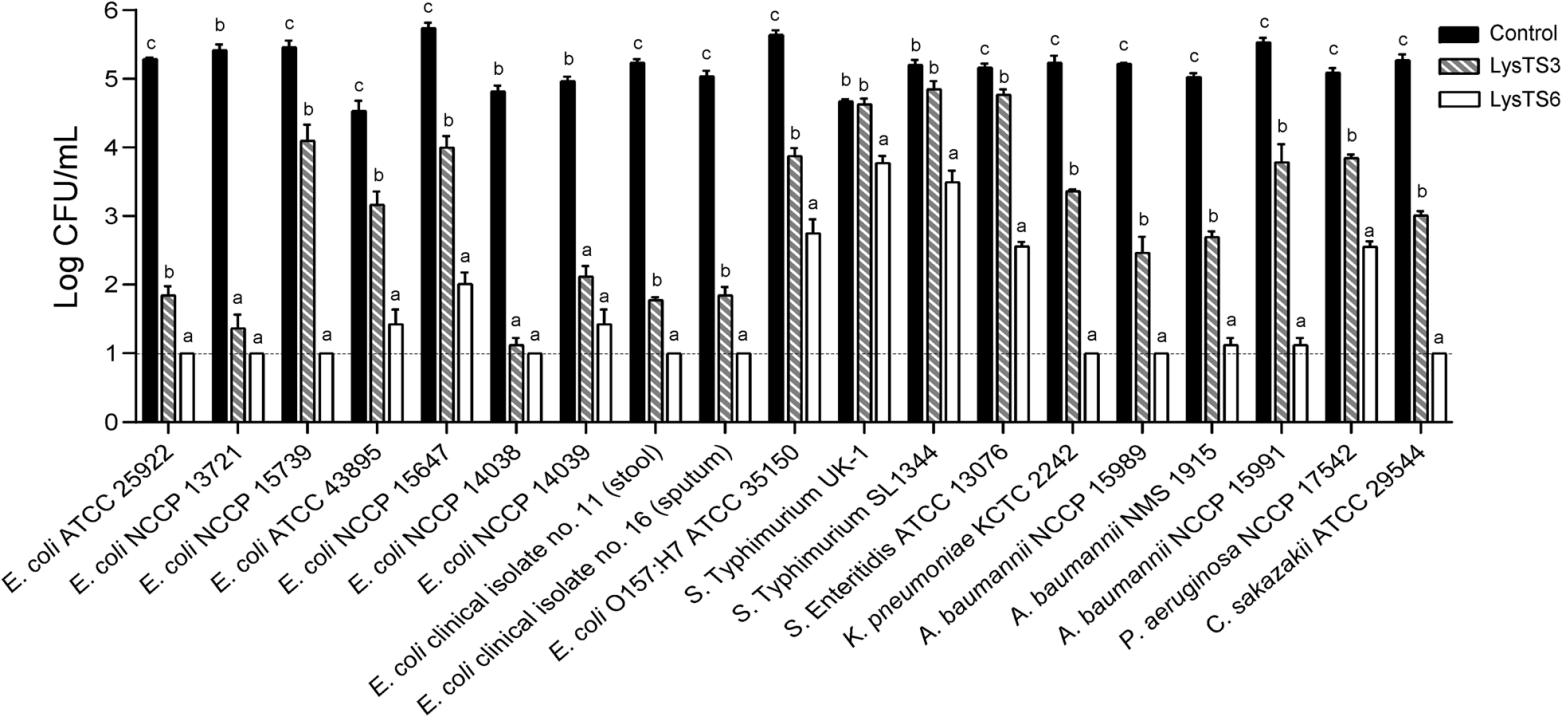
Antibacterial spectrum of LysTS3 and LysTS6 against various Gram-negative bacteria without membrane permeabilization. Exponentially grown Gram-negative bacteria including *E. coli*, *Salmonella, K. pneumoniae*, *A. baumannii*, *P. aeruginosa* and *C. sakazakii* were incubated with 1 μM of each endolysin at 37 °C for 1 h and CFUs were measured. The endolysin storage buffer served as a negative control. All experiments were performed in triplicate. Data represent mean ± standard deviation and the dotted horizontal lines represent the limit of detection. Statistical significance was analyzed by one-way analysis of variance (ANOVA) with Tukey’s multiple-comparison test among the experimental groups. The different letters indicate statistically significant differences

Antibacterial activities of LysTS3 and LysTS6 were compared further against the *E. coli* without membrane-permeabilization. Interestingly, the endolysins showed significant differences in antibacterial activities against *E. coli* despite having similar muralytic activities against the membrane-permeabilized *E. coli* (Table S3). When *E. coli* cells were treated with the endolysins for 1 h without membrane-permeabilization, the antibacterial activity of LysTS6 was significantly higher than that of LysTS3 at concentrations above 0.1 μM (Fig. 1A). One μM of LysTS3 reduced viable *E. coli* counts up to 4.1 logs after 2 h of treatment, however, the same concentration of LysTS6 was able to show 4 logs reduction within 15 min and eliminated *E. coli* to undetectable level after 30 min of treatment (Fig. 1B). Membrane damages of *E. coli* by LysTS3 or LysTS6 were observed using SEM. Both LysTS3 and LysTS6 could cause *E. coli* membrane damage, but LysTS6 produced much higher membrane damage compared with LysTS3 (Fig. S3). The antibacterial spectrum of LysTS3 and LysTS6 endolysins was evaluated against various Gram-negative bacteria, including several multidrug-resistant (MDR) strains (Fig. 2). Strains used in this study and their antibiotic resistance information are listed in Table S1. As shown in Fig. 2, LysTS6 generally showed stronger antibacterial activity compared to LysTS3 against most Gram-negative bacteria tested. The antibacterial activity of LysTS3 was notably low against three different *Salmonella* strains tested.

### The C-terminal amphipathic helix is crucial for differential antibacterial activity between LysTS3 and LysTS6

We established that LysTS6 had superior antibacterial activity against Gram-negative bacteria than LysTS3 despite their overall similarities in sequences, structures, and muralytic activities. To elucidate mechanisms accountable for their differential antibacterial activities against Gram-negative bacteria, we analyzed the differences in the amino acid residues of these two endolysins other than the N-terminal domain (NTD) because both endolysins have glycosidase domains at the N-terminus. The C-terminal domains (CTDs) of LysTS3 (amino acid residues from 82 to 162) and LysTS6 (amino acid residues from 80 to 160) have amphipathic helices similar to those in T4 lysozyme. Klaus Düring et al. [36] previously reported that the C-terminal amphipathic helices (amino acid residues from 126 to 155) of T4 lysozyme could facilitate the penetration of the membrane of Gram-negative bacteria and fungi. Structural analysis revealed that amphipathic helices similar to these membrane-penetrating helices of T4 lysozyme were found in LysTS3 (amino acid residues from 133 to 162) and LysTS6 (amino acid residues from 131 to 160) at the C-terminus (Fig. S4). These C-terminal amphipathic helices of both endolysins consisting of two amphipathic helices, α1 (amino acid residues from 133 to 147 in LysTS3 and amino acid residues from 131 to 145 in LysTS6) and α2 (amino acid residues from 151 to 162 in LysTS3 and amino acids residues from 149 to 160 in LysTS6), showing high sequence similarity with each other (80% identity). However, two amino acid residues in α1 helix and four amino acid residues in α2 helix were different between LysTS3 and LysTS6 (Fig. S1A) that roles of α1 and α2 helices on endolysin activity were tested by making chimeric endolysins of LysTS3 and LysTS6.

Three chimeric endolysins were generated by fusing the amino acid residues from 1 to 81 (LysTS3ΔCTD), the amino acid residues from 1 to 131 [LysTS3Δ(α1–α2)], and the amino acid residues from 1 to 147 (LysTS3Δα2) of LysTS3 with the fragments from LysTS6 corresponding to the deleted fragments of LysTS3 (Fig. S5A). When comparing their antibacterial activities against *E. coli*, all three chimeric endolysins showed significantly greater antibacterial activity compared to LysTS3. It is interesting to note that even the chimeric endolysin of LysTS3 replaced with the C-terminal α2 helix from LysTS6 consisting of only 12 amino acid residues showed high antibacterial activity comparable to LysTS6 (Fig. S5B). These results led us to hypothesize that the key factor determining the differences in their antibacterial activity against Gram-negative bacteria may exist on the α2 helix of these endolysins. Further analysis was focused on the 4 amino acid residues that are different between the C-terminal α2 helices of LysTS3 and LysTS6.

### Differences in the surface charge on the C-terminal α2 helices of LysTS3 and LysTS6

**Fig. 3:**
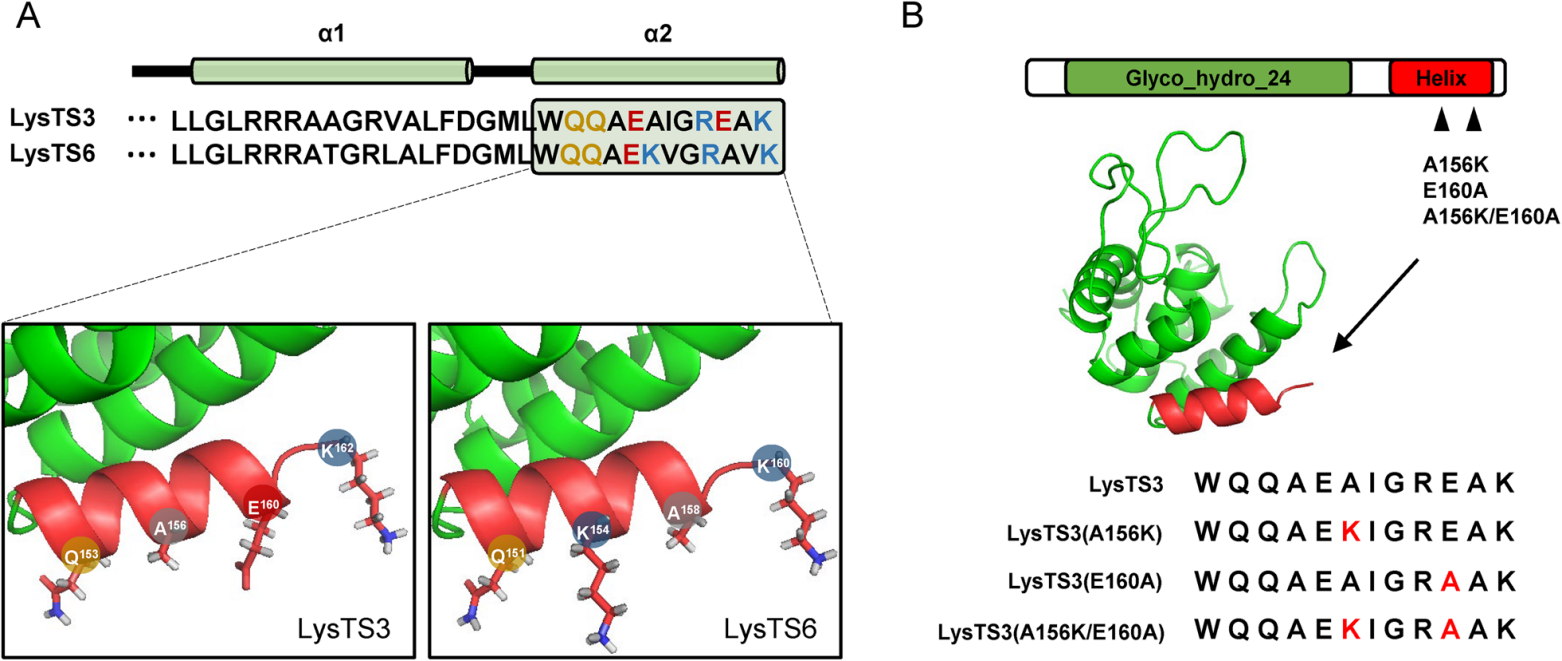
Scheme for the generation of LysTS3 mutant endolysins through targeted point mutations. **A** Schematic representation of the charged amino acid residues on the surface of the C-terminal α2 helix of two endolysins. Amino acid residues in the C-terminal α2 helix are represented in different colors depending on the chemical natures of their side chains: black, nonpolar amino acid residues; yellow, polar amino acid residues; red, negatively charged amino acid residues; and blue, positively charged amino acid residues. **B** Scheme of the point mutations of LysTS3. Single mutants were generated by replacing the nonpolar amino acid Ala at amino acid residue 156 and the negatively charged amino acid Glu at amino acid residue 160 with lysine and alanine, respectively. The double mutant of LysTS3 was generated by substituting both Ala_156_ and Glu_160_ with lysine and alanine

Structural analysis of the C-terminal α2 helices of LysTS3 and LysTS6 revealed that 2 out of the 4 amino acid residues that differ between the C-terminal α2 helices of both endolysin are located on the surface of the helix (Fig. 3A). The surface-exposed residues on the C-terminal α2 helix of LysTS3 comprised of Gln_153_, Ala_156_, Glu_160_, and Lys_162_, which are nonpolar and negatively charged amino acid residues. In contrast, the corresponding amino acid residues on the C-terminal α2 helix of LysTS6 are Gln_151_, Lys_154_, Ala_158_, and Lys_160_, making the surface charge more positive compared to LysTS3 (Fig. 3A). The positive charge of the surface on the amphipathic helix would enhance interaction with the negatively charged outer membrane of Gram-negative bacteria [37]. To test if the differences in surface charge between the C-terminal α2 helices of the two endolysins directly contribute to their differential antibacterial activity, we generated point mutations at 156 and 160 amino acid residues of LysTS3 located on the surface of the α2 helix, in which amino acid residues have charge differences between the two endolysins. Three mutants including a mutant containing Ala_156_ to lysine substitution (A156K), a mutant containing Glu_160_ to alanine substitution (E160A), and a mutant containing both amino acid residue substitutions (A156K/E160A) were constructed (Fig. 3B).

### Effect of the surface charge of the C-terminal amphipathic helix on the differential antibacterial activity of LysTS3 and LysTS6

**Fig. 4:**
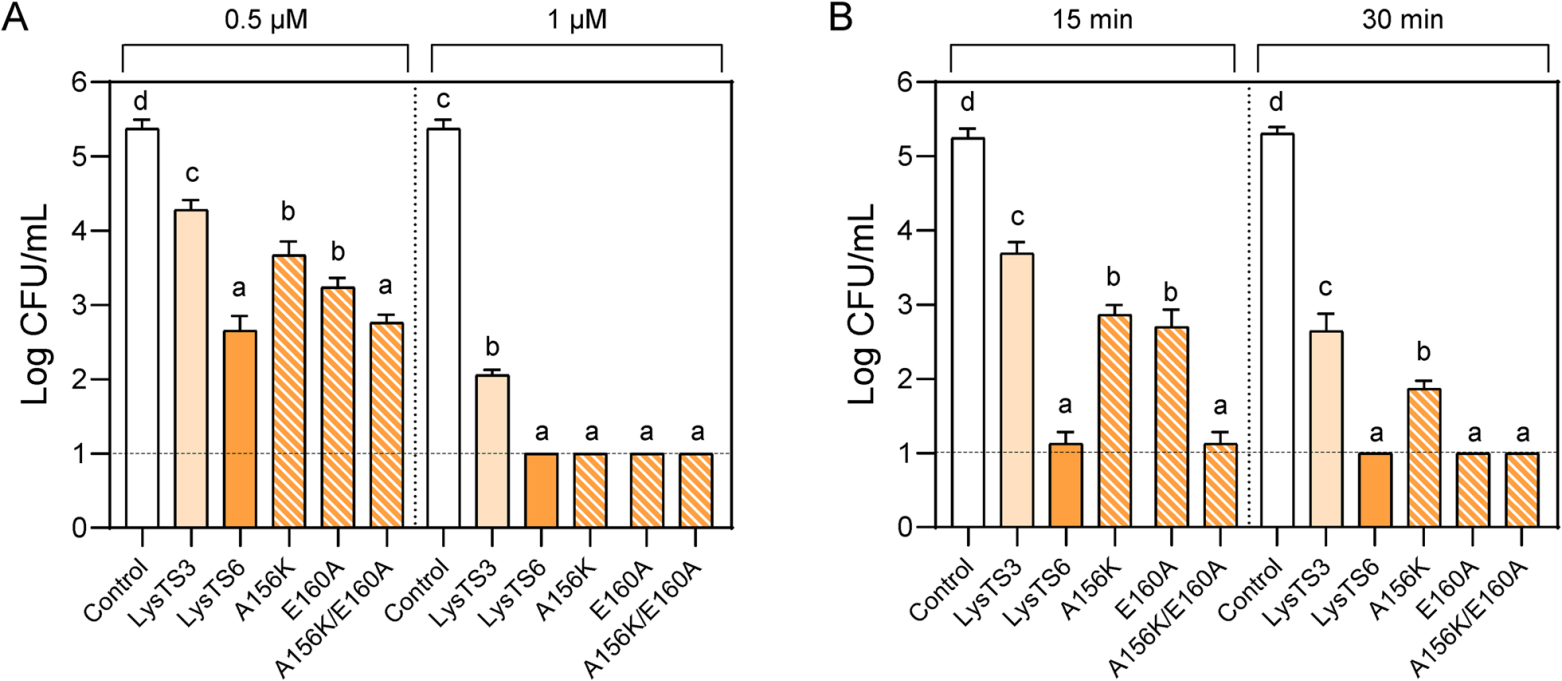
Surface charge of the C-terminal α2 helix influences the antibacterial activity of LysTS3. **A** Antibacterial activity of LysTS3, LysTS6, and three LysTS3 mutants (A156K, E160A, and A156K/E160A) at different concentrations. Exponential phase *E. coli* were treated with 0.5 μM or 1 μM of endolysins, incubated at 37 °C for 1 h, and CFUs were measured. The endolysin storage buffer served as a negative control. **B** Antibacterial activity of LysTS3, LysTS6, and three LysTS3 mutants (A156K, E160A, and A156K/E160A) against *E. coli* at different time points. Exponential phase *E. coli* were treated with 1 μM of endolysins, incubated at 37 °C, and CFUs were measured after 15 min and 30 min. The endolysin storage buffer served as a negative control. All experiments were performed in triplicate. Data represent mean ± standard deviation and the dotted horizontal lines represent the limit of detection. Statistical significance was analyzed by one-way analysis of variance (ANOVA) with Tukey’s multiple-comparison test among the experimental groups. The different letters indicate statistically significant differences

The antibacterial activities of LysTS3, LysTS6, and LysTS3 mutants with amino acid residue substitutions were evaluated against *E. coli* at concentrations of 0.5 μM and 1 μM (Fig. 4) because differences in the antibacterial activities of LysTS3 and LysTS6 against *E. coli* were large enough for easy comparison at these concentrations (Fig. 1A)*.* When *E. coli* were treated with endolysins at 0.5 μM and 1 μM for 1 h, all LysTS3 mutants and LysTS6 demonstrated significantly higher antibacterial activity compared to LysTS3 (Fig. 4A). LysTS6 and the A156K/E160A mutant demonstrated the highest antibacterial activity at 0.5 μM. At 1 μM, all endolysins except LysTS3 reduced bacterial counts to undetectable level within 1 h, making an accurate comparison difficult. To further evaluate the antibacterial activity of endolysins at 1 μM, we assessed the reduction in bacterial counts at 15 min and 30 min (Fig. 4B). LysTS6 and A156K/E160A exhibited the highest antibacterial activity with a log reduction of 4.13 and A156K and E160A mutants showed log reductions of 2.38 and 2.67, respectively, after 15 min of treatment. LysTS3 showed the lowest activity with a log reduction of 1.56 after 15 min of treatment. After 30 min of treatment, LysTS6 and all mutants showed significantly higher antibacterial activity than LysTS3 and LysTS6, E160A, and A156K/E160A reduced bacterial counts to undetectable level. These results indicate that each single mutation can increase LysTS3 activity and double mutation showed additive effects on LysTS3 activity against Gram-negative bacteria.

The activities of endolysins were also accessed with membrane permeability using NPN and SYTOX uptake assays. NPN detects damage to the outer membrane of Gram-negative bacteria by emitting fluorescence when entering the periplasm, whereas SYTOX detects damage to the inner membrane by fluorescing upon binding to DNA. The highest uptake of NPN was observed when 1 µM of LysTS6 or A156K/E160A were applied whereas treatment with the same amount of LysTS3 resulted in the lowest uptake of NPN (Fig. S6A), suggesting that the C-terminal α2 helix with the positive surface charge present at LysTS6 and A156K/E160A could make *E. coli* outer membrane more permeable to endolysin. This can help LysTS6 and A156K/E160A to access the peptidoglycan layer, making these enzymes have the highest antibacterial activity. The SYTOX assay results indicate that endolysins showing high outer membrane permeability exhibited high inner membrane damage (Fig. S6B). These results suggest that the membrane permeability of the endolysin may be governed by the surface charge on the C-terminal α2 helix of the endolysin.

As shown in Fig. S1A and Fig. 3A, the differences in the amino acid residues of the two endolysins were also observed at the positions Ile_157_ and Ala_161_ in LysTS3, corresponding to Val_155_ and Val_159_ in LysTS6. These residues are located inside the C-terminal α2 helix and two LysTS3 mutants containing I157V or A161V substitutions did not show significant differences in the antibacterial activity against *E. coli* compared to LysTS3 (Fig. S7). The LysTS6 mutant (K154A/A158E) in which Lys_154_ and Ala_158_ on the C-terminal amphipathic helix were substituted with alanine and glutamate exhibited reduced antibacterial activity similar to that of LysTS3 against *E. coli* (Fig. S8). These results demonstrate that the difference in the antibacterial activity between LysTS3 and LysTS6 against Gram-negative bacteria is primarily due to the charge differences on the surface of the C-terminal α2 helix.

### Enhancement of antibacterial activity through modification of the surface charge on the C-terminal amphipathic helices of other endolysins

**Fig. 5:**
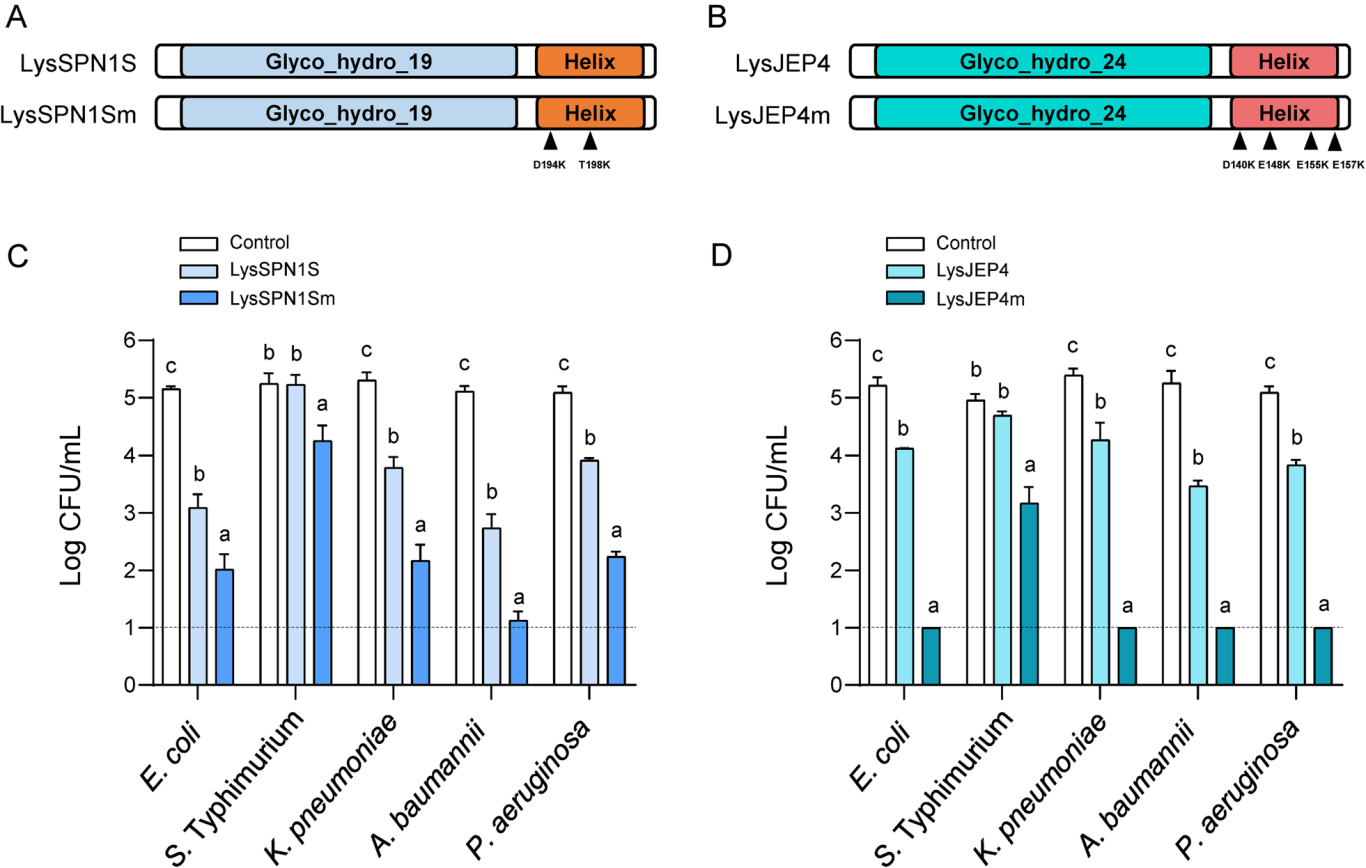
Modification of surface charge in the C-terminal helix enhanced antibacterial activities of LysSPN1S and LysJEP4. **A** Schematic representation of LysSPN1S and LysSPN1Sm. LysSPN1Sm was constructed by replacing Asp_194_ and Thr_198_ on the surface of the C-terminal amphipathic helix of LysSPN1S with lysine. The positions of point mutations (D194K and T198K) are indicated by triangles. **B** Schematic representation of LysJEP4 and LysJEP4m. LysJEP4m was constructed by replacing Asp_140_, Glu_148_, Glu_155_, and Glu_157_ on the surface of the C-terminal amphipathic helix of LysJEP4 with lysine. The positions of point mutation (D140K, E148K, E155K, and E157K) are indicated by triangles. **C** Antibacterial activity of LysSPN1S and LysSPN1Sm against Gram-negative bacteria. **D** Antibacterial activity of LysJEP4 and LysJEP4m against Gram-negative bacteria. Exponential phase Gram-negative bacteria were incubated with 1 μM of each endolysin at 37 °C for 1 h and CFUs were measured. The endolysin storage buffer served as a negative control. The experiments were repeated in triplicate. Data represent mean ± standard deviation and the dotted horizontal lines represent the limit of detection. Statistical significance was analyzed by one-way analysis of variance (ANOVA) with Tukey’s multiple-comparison test among the experimental groups. The different letters indicate statistically significant differences

The importance of the surface charge of the C-terminal amphipathic helix of endolysin was tested to other endolysins containing the C-terminal amphipathic helices. Structural analysis of several endolysins available in our laboratory revealed that LysSPN1S and LysJEP4 also possess C-terminal amphipathic helix (Fig. S9). The surface on the C-terminal amphipathic helix of LysSPN1S consisting of the amino acid residues from 191 to 207 contains Asp_194_ along with Thr_198_ (Fig. S10A) and the surface on the C-terminal amphipathic helix of LysJEP4 consisting of the amino acid residues from 139 to 158 contains negatively charged amino acid residues, Asp_140_, Glu_148_, Glu_155_, and Glu_157_ (Fig. S10B). We generated LysSPN1Sm by replacing the Asp_194_ and Thr_198_ with lysine to increase the positive charge on the surface of the C-terminal amphipathic helix (Figs. 5A and S11A). Similarly, we generated LysJEP4m by replacing Asp_140_, Glu_148_, Glu_155_, and Glu_157_ with lysine on the surface of the C-terminal amphipathic helix (Figs. 5B and S11A). As shown in Fig. S11B, the muralytic activities of LysSPN1S and LysSPN1Sm were almost same, and LysJEP4m retained above 80% of the muralytic activity of LysJEP4 when tested against the membrane-permeabilized *E. coli*. When 1 μM of the endolysins were tested against various Gram-negative bacteria, LysSPN1Sm reduced *E. coli* by 3.38 logs, *S.* Typhimurium by 1.05 logs, *K. pneumoniae* by 3.23 logs, *A. baumannii* by 4.01 logs, and *P. aerugniosa* by 2.84 logs. In contrast, LysSPN1S reduced *E. coli* by 2.14 logs, *K. pneumoniae* by 1.56 logs, *A. baumannii* by 2.45 logs, *P. aerugniosa* by 1.16 logs but it had no effect on *S*. Typhimurium (Fig. 5C). LysJEP4m completely eradicated all tested bacterial strains with the exception of *S*. Typhimurium to undetectable level, whereas LysJEP4 reduced *E. coli* by 1.06 logs, *K. pneumoniae* by 1.27 logs, *A. baumannii* by 1.73 logs, and *P. aerugniosa* by 1.25 logs. LysJEP4m also reduced *S.* Typhimurium by 1.92 logs, but LyJEP4 had no effect on *S*. Typhimurium (Fig. 5D). These results indicate that modifying surface charge on the C-terminal amphipathic helix to be more positive could significantly enhance their antibacterial activities against Gram-negative bacteria.

## Discussion

Antibiotic-resistant Gram-negative bacteria hinder efficient treatment of infection, elevating the risk of patient mortality [3–8]. Despite the continuous development of new antibiotics, constant efforts for discovery of alternatives to antibiotics are still required to solve this problem. Endolysins have garnered significant attention as a promising alternative to conventional antibiotics, but they cannot be applied to Gram-negative bacteria due to the outer membrane. Recently, some endolysins featuring amphipathic helices at the C-terminus have been reported to have intrinsic antibacterial activity against Gram-negative bacteria [20–30]. As more of these endolysins are being discovered, a deeper understanding of them is required to optimize and further advance their utilization.

In this study, we identified two glycoside hydrolase endolysins, LysTS3 and LysTS6, that have high similarity in amino acid sequences and structures with each other (Fig. S1). Both endolysins exhibited antibacterial activity against Gram-negative bacteria without membrane-permeabilization (Figs. 1 and 2) due to their amphipathic helices in the CTD (Fig. S4), which could allow them to penetrate the outer membrane of Gram-negative bacteria and access their peptidoglycan layer. No differences were observed in their muralytic activity against membrane-permeabilized *E. coli* (Table S3) but LysTS6 showed higher antibacterial activity and membrane permeability than LysTS3 against Gram-negative bacteria without membrane-permeabilization (Figs. 1, 2 and Figs. S3, S6). These results suggest that their differential antibacterial activity is likely due to differences in the amino acid residues other than the NTD that has an enzymatically active domain.

The C-terminal helix (amino acid residues from 143 to 155) of T4 lysozyme is the main factor in determining its antibacterial activity against Gram-negative bacteria. In particular, the positively charged amino acid residues within its helix are known to play a crucial role in controlling Gram-negative bacteria by facilitating interactions with the negatively charged outer membrane. Similarly, our data showed that the C-terminal α2 helices of LysTS3 and LysTS6 were responsible for their differential antibacterial activity (Fig. S5) and the positive surface charge on the the C-terminal α2 helix was identified as the main determinant of the antibacterial activity and membrane permeability of the endolysins against Gram-negative bacteria (Fig. 4 and Figs. S6–8). It is noteworthy that each single mutation which increased the positive surface charge by substituting the amino acid residues Ala_156_ or Glu_160_ in the C-terminal α2 helix of LysTS3 with lysine or alanine of LysTS6 resulted in improved antibacterial activity and membrane permeability than LysTS3 and that the double mutation where both amino acid residues were replaced exhibited an additive effect, resulting in high activity comparable to LysTS6 (Fig. 4 and Figs. S6–8). Our data also showed that 1 μM of E160A in which the negatively charged 160th glutamic acid of LysTS3 was replaced by alanine exhibited higher antibacterial activity after 30 min of reaction compared to 1 μM of A156K where 156th alanine of LysTS3 was replaced by lysine (Fig. 4B), suggesting that the removal of the negatively charged amino acid residues exposed on the surface of the C-terminal amphipathic helix in endolysins might have a significant effect on improving antibacterial activity of endolysins. These observations underscore the importance of the positive charge on the surface of the C-terminal amphipathic helix as a key factor underlying the antibacterial activity of the endolysins against Gram-negative bacteria and suggest that the positive charges on the surface of the C-terminal amphipathic helix of endolysin may enhance interaction with the negatively charged outer membrane through stronger electrostatic interactions and facilitate outer membrane penetration by endolysin. Building on these observations, we applied our approach to the other two endolysins, LysSPN1S and LysJEP4, which also possess C-terminal amphipathic helices. When increasing the positive charge on the surface of the C-terminal amphipathic helices of the LysSPN1S and LysJEP4 through site-directed mutagenesis, the mutants exhibited substantially improved antibacterial activities compared to the native endolysins while retaining most of the muralytic activities of their wild-type endolysins (Figs. 5 and S11). These results provide strong evidence that our approach can be applied across different endolysins with C-terminal amphipathic helices and that surface charge modification could be utilized to optimize the antibacterial activities of these endolysins.

Several studies have explored the antibacterial activity of endolysins containing C-terminal amphipathic helices [20–30], but their practical application has been limited due to their low activities. Shih-Yi Peng et al. [38] have demonstrated that introduction of positively charged amino acid residues into the hydrophilic face of the peptide consisting of the C-terminal amphipathic helix (amino acid residues from 113 to 145) of LysAB2 enhanced antibacterial activity of the peptide against Gram-negative bacteria. However, their work studied the effects of surface charge on the antibacterial activity of the peptide itself but not on the antibacterial activity of endolysin containing the C-terminal amphipathic helix against Gram-negative bacteria. In this study, we introduced point mutations directly into the C-terminal helices of endolysins. We introduced point mutations into the C-terminal helix of the endolysins. The muralytic activity of each mutant retained more than 80% of the activity observed in the wild-type endolysin (Fig. S11 and Table S3) and their predicted structures were almost identical to those of the wild-type endolysins (Fig. S12), suggesting that the mutations did not cause significant changes to their structure or polymerization status. Further structural investigations should aim to clarify the effect of the surface charge modification on conformational and polymerization changes in endolysin. We also experimentally demonstrated that surface charge is a key determinant of antibacterial activity in endolysin containing C-terminal amphipathic helix and that modification of the surface charge on the C-terminal helix to be more positive can boost the antibacterial activity of the endolysin. To the best of our knowledge, this is the first study to demonstrate that the surface charge of the C-terminal amphipathic helix plays a crucial role in the antibacterial activity of endolysins. Our study cannot entirely rule out the possibilities that other factors may also influence the antibacterial activity of endolysin, but these results provide valuable insights into the role of surface charge in endolysins with C-terminal amphipathic helices and hold significant implications for maximizing the activity of endolysins containing the C-terminal amphipathic helix or discovering novel endolysins with high potency to control Gram-negative bacteria.

## Conclusions

This study investigated the role of surface charges on the C-terminal amphipathic helix of endolysin on the antibacterial activity against Gram-negative bacteria. Despite the high similarities in amino acid residues and muralytic activity, LysTS6 demonstrated greater antibacterial activity against various Gram-negative bacteria than LysTS3. Systematic truncation analysis of LysTS3 and LysTS6 revealed that the main cause of the differences in antibacterial activity between LysTS3 and LysTS6 against Gram-negative bacteria existed on the C-terminal α2 helix of these endolysins and bioinformatics analysis revealed that the C-terminal α2 helix of LysTS6 had more positive surface charge than that of LysTS3. Each single mutation that increased the positive charge by replacing amino acid residues of LysTS3 at positions 156 and 160, where charge differences exist between the C-terminal α2 helix of two endolysins, with lysine and alanine from LysTS6 resulted in higher antibacterial activity and membrane permeability than LysTS3 and the double mutation at positions 156 and 160 exhibited an additive effect, resulting in high antibacterial activity and membrane permeability comparable to LysTS6. Similar activity improvement against Gram-negative bacteria was also possible with LysSPN1S and LysJEP4 by increasing the positive charge on the C-terminal helix of LysSPN1S and LysJEP4 through site-directed mutagenesis. These findings emphasize the importance of the surface charge on the C-terminal helix and suggest the potential of a novel endolysin engineering approach through modifications of the surface-exposed amino acid residues on the C-terminal helix of endolysin. Our study holds considerable promise for the future advancement and applications of endolysins to combat Gram-negative bacteria.

## Supplementary Information

Supplymentary materilas 1.

## Data Availability

The complete genome sequences of phage TS3, TS6, JEP4, and SPN1S were deposited in NCBI’s Genbank and are available via accession number MK249126, NC_069148, MT740315, and NC_016761, respectively. The accession numbers for endolysin LysTS3, LysTS6, LysJEP4 and LysSPN1S are AZU99464, YP_010582290, QOC67906, and YP_005098003, respectively. The data sets reported in this study will be accessible from the corresponding author upon reasonable request.

## Abbreviations

WHO: World Health Organization
LPS: Lipopolysaccharide
LB: Luria–Bertani
Km: Kanamycin
IPTG: Isopropyl β-d-thiogalactopyranoside
PCR: Polymerase chain reaction
OD: Optical density
Ni–NTA: Nickel-nitrilotriacetic acid
SDS-PAGE: Sodium dodecyl sulfate–polyacrylamide gel electrophoresis
SEM: Scanning electron microscope
PBS: Phosphate-buffered saline
NPN: N-phenyl-1-napthylamine
RFU: Relative fluorescence units
ORF: Open reading frame
EDTA: Ethylenediaminetetraacetic acid
NTD: N-terminal domain
CTD: C-terminal domain
