# Supplementary material for: Surface charge of the C-terminal helix is crucial for antibacterial activity of endolysin against Gram-negative bacteria
Source: J Biomed Sci. 2025 Mar 22;32:38. doi: 10.1186/s12929-025-01133-x (PMC11929351; doi:10.1186/s12929-025-01133-x)
Supplement: Supplementary file 1 — Supplymentary materilas 1. [file 12929_2025_1133_MOESM1_ESM.pdf]

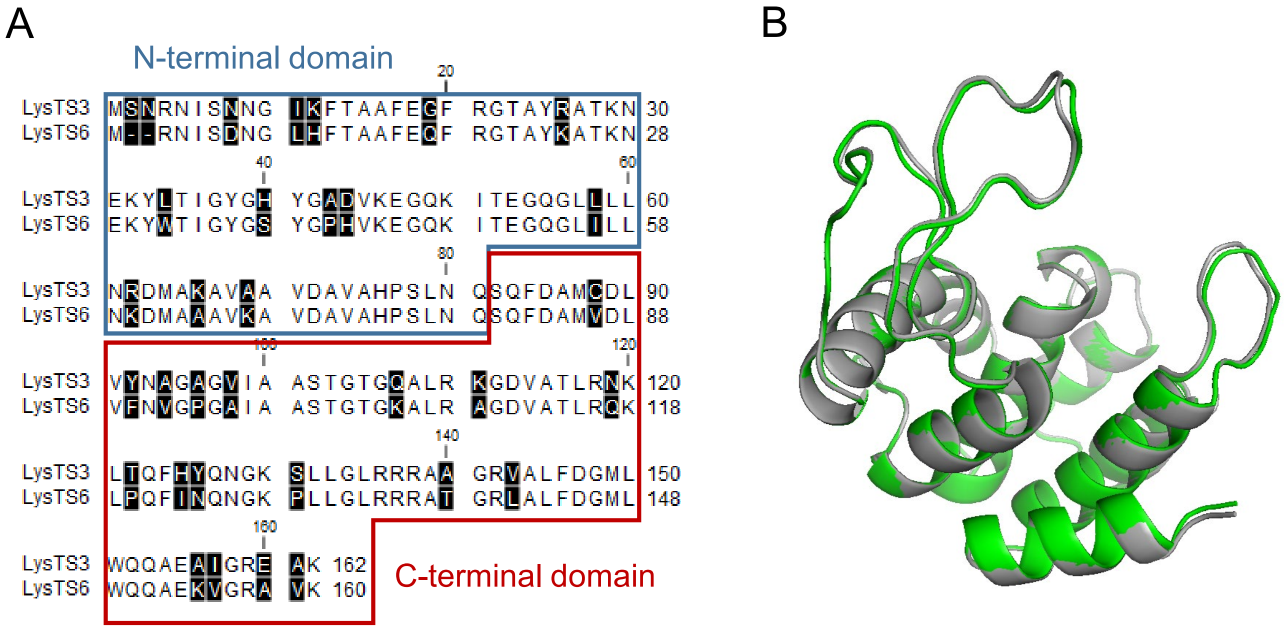

Figure S1. Comparison of amino acid sequences and structures of LysTS3 and LysTS6 endolysins. (A) Alignment of amino acid sequences of LysTS3 and LysTS6. Different amino acid residues between LysTS3 and LysTS6 are shaded in black. The N-terminal domain and C-terminal domain are marked with blue and red boxes, respectively. (B) Structure alignment of LysTS3 and LysTS6. The three-dimensional structures of LysTS3 and LysTS6 were predicted by Alphafold-3 and aligned with each other. The predicted structure of LysTS3 (green) exhibited a high degree of similarity to that of LysTS6 (grey).

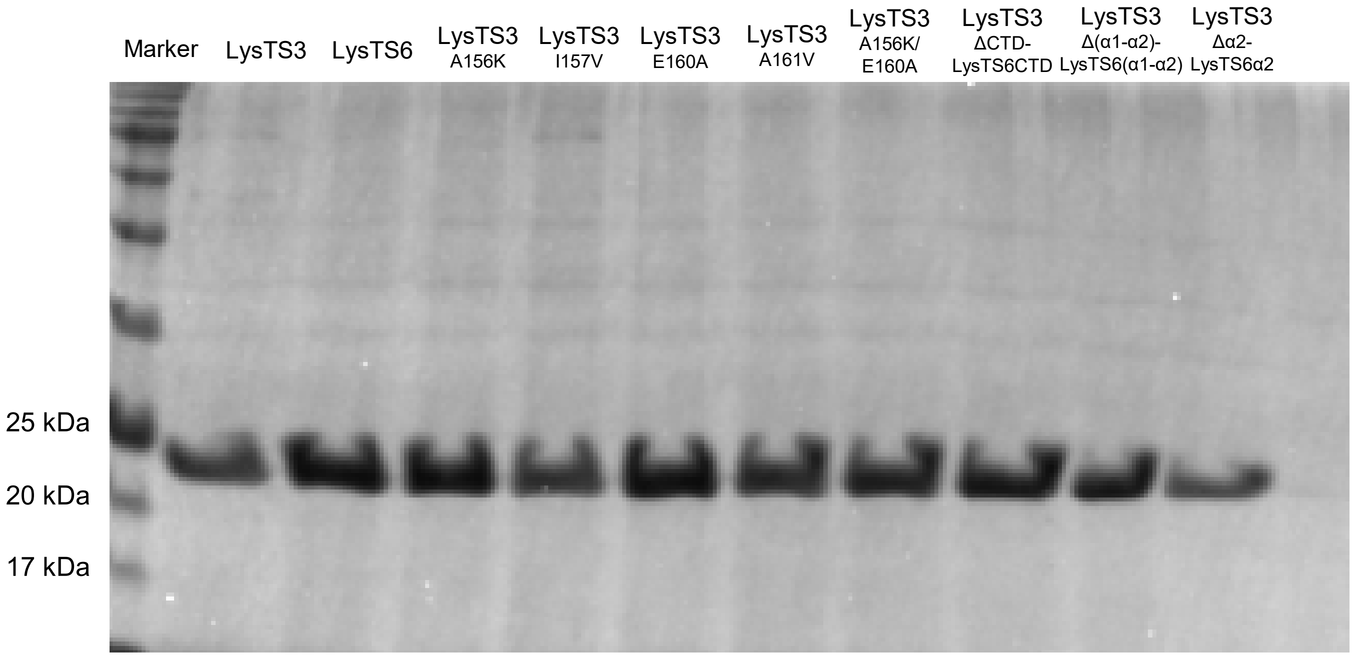

Figure S2. SDS-PAGE analysis of LysTS3, LysTS6, and LysTS3 mutants. Purified LysTS3, LysTS6, and various mutants of LysTS3 used in this study were analyzed on 15% SDS-PAGE gel.

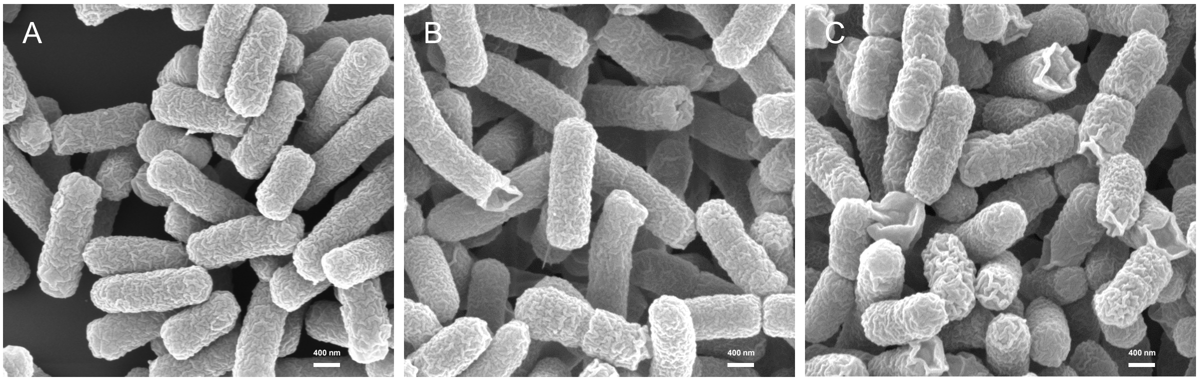

Figure S3. Scanning electron microscopy of *E. coli* ATCC 25922. *E. coli* ATCC 25922 were treated with storage buffer (A), LysTS3 (B), and LysTS6 (C) for 15 min and SEM pictures were taken.

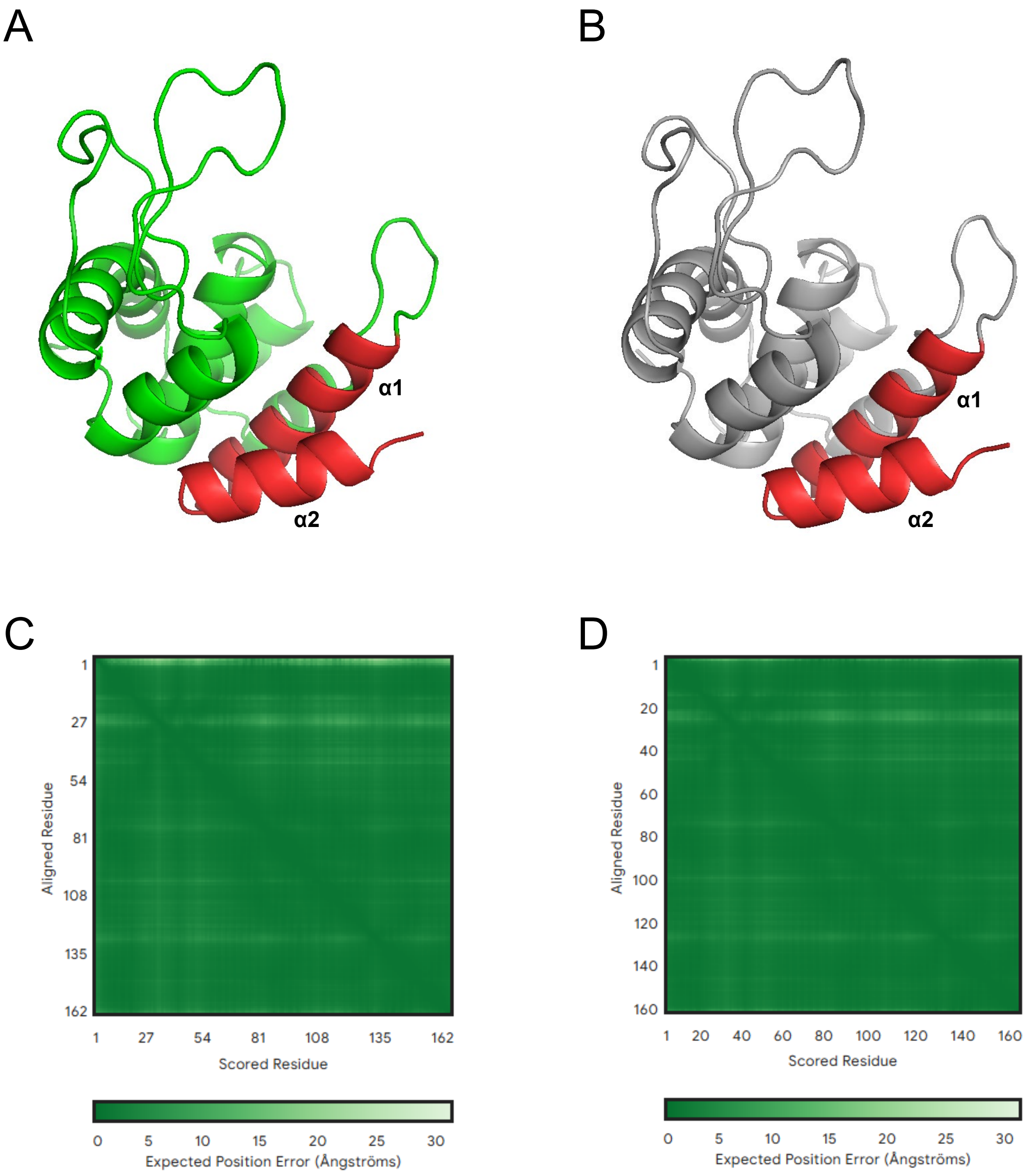

Figure S4. Predicted three-dimensional structures of the C-terminal amphipathic helices of LysTS3 and LysTS6 by Alphafold-3. The three-dimensional structures of LysTS3 (A) and LysTS6 (B) were predicted by Alphafold-3. The C-terminals of LysTS3 (amino acid residues from 133 to 162) and LysTS6 (amino acid residues from 131 to 160) have amphipathic helix structures similar to the membrane-penetrating peptides of T4 lysozyme and are shown in red color. These C-terminal amphipathic helices of both endolysins consist of two amphipathic helices, α1 (amino acid residues from 133 to 147 in LysTS3 and amino acid residues from 131 to 145 in LysTS6) and α2 (amino acid residues from 151 to 162 in LysTS3 and amino acids residues from 149 to 160 in LysTS6). The Predicted Aligned Error (PAE) plots represent the structural confidence of the protein prediction models for LysTS3 (C) and LysTS6 (D).

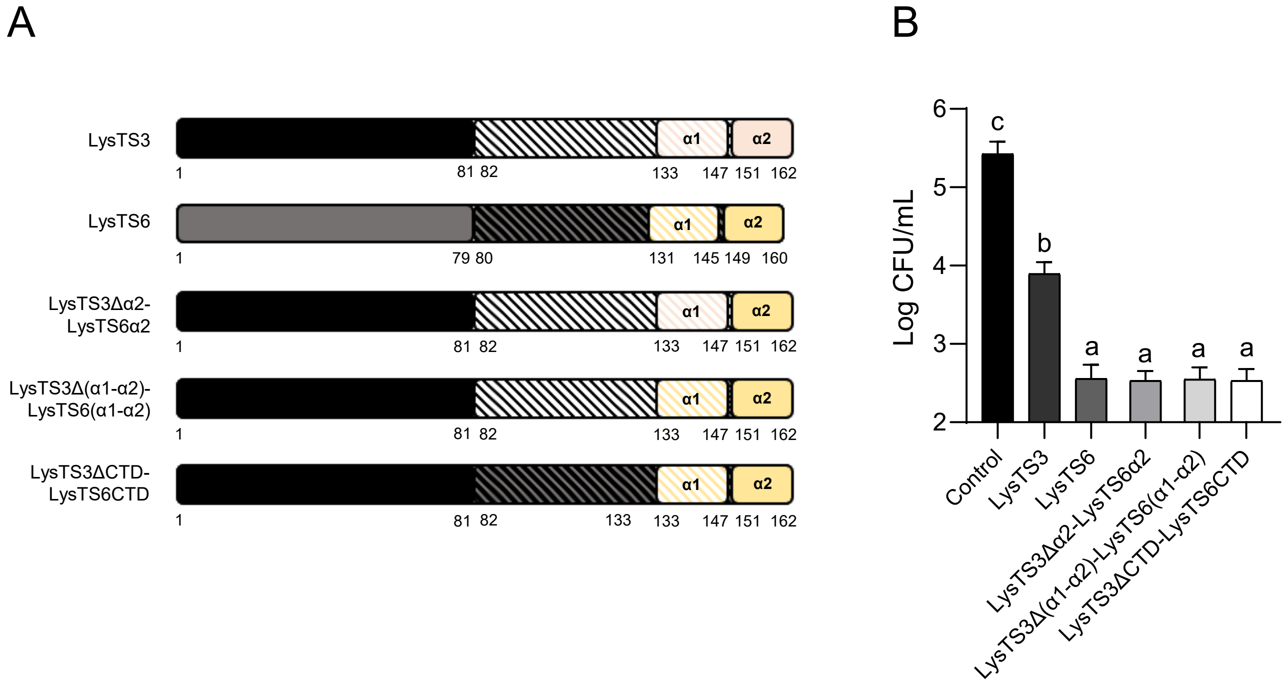

Figure S5. Schematic representation of chimeric endolysins and their antibacterial activity against *E. coli*. (A) Schematic representation of LysTS3, LysTS6, LysTS3Δα2-LysTS6α2, LysTS3Δ(α1-α2)-LysTS6(α1-α2), LysTS3ΔCTD-LysTS6CTD. black box, LysTS3NTD; gray box, LysTS6NTD; white diagonal stripe box, LysTS3CTD; gray diagonal stripe box, LysTS6CTD; pink diagonal stripe box, LysTS3α1; pink box, LysTS3α2; yellow diagonal stripe box, LysTS6α1; yellow box, LysTS6α2. (B) Antibacterial activity of the chimeric endolysins against *E. coli*. Exponentially grown *E. coli* were resuspended in the buffer (20 mM HEPES, pH 7.4) and were treated with 500 nM of the endolysins. After incubation at 37°C for 1 h, the serially diluted cells were spotted onto the LB agar and enumerated. Error bars indicate standard deviation, and data are replicated for three independent experiments. Statistical significance was analyzed by one-way analysis of variance (ANOVA) with Tukey's multiple-comparison test among the experimental groups. The different letters indicate statistically significant differences.

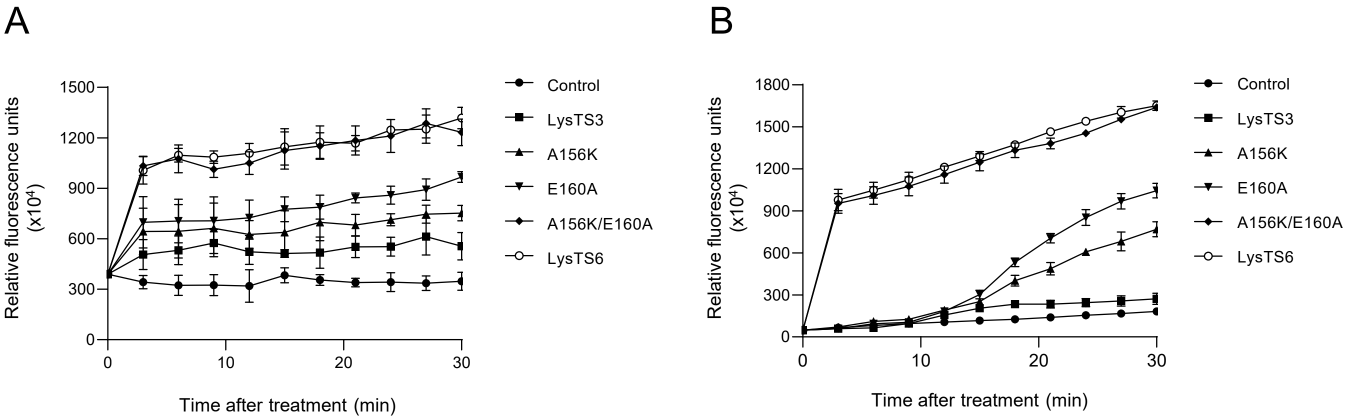

Figure S6. Membrane permeability of LysTS3, LysTS6, and LysTS3 mutants. Two different methods were used for the membrane permeability assay; the NPN uptake assay for the outer membrane permeability and SYTOX assay for the cell membrane permeability. (A) Determination of outer membrane permeability using NPN uptake assay. Exponentially grown *E. coli* ATCC 25922 were treated with 1 μM of the endolysins and 10 μM of NPN and incubated at 37°C for 30 min. The fluorescence was measured 3 min intervals up to 30 min using a spectrophotometer with an excitation wavelength of 350 nm and an emission wavelength of 420 nm. (B) Determination of cell membrane permeability using SYTOX assay. Exponentially grown *E. coli* ATCC 25922 were treated with 1 μM of the endolysins and 1 μM of SYTOX and incubated at 37°C for 30 min. The fluorescence was measured 3 min intervals up to 30 min using a spectrophotometer with an excitation wavelength of 488 nm and an emission wavelength of 522 nm. All experiments were repeated in triplicate.

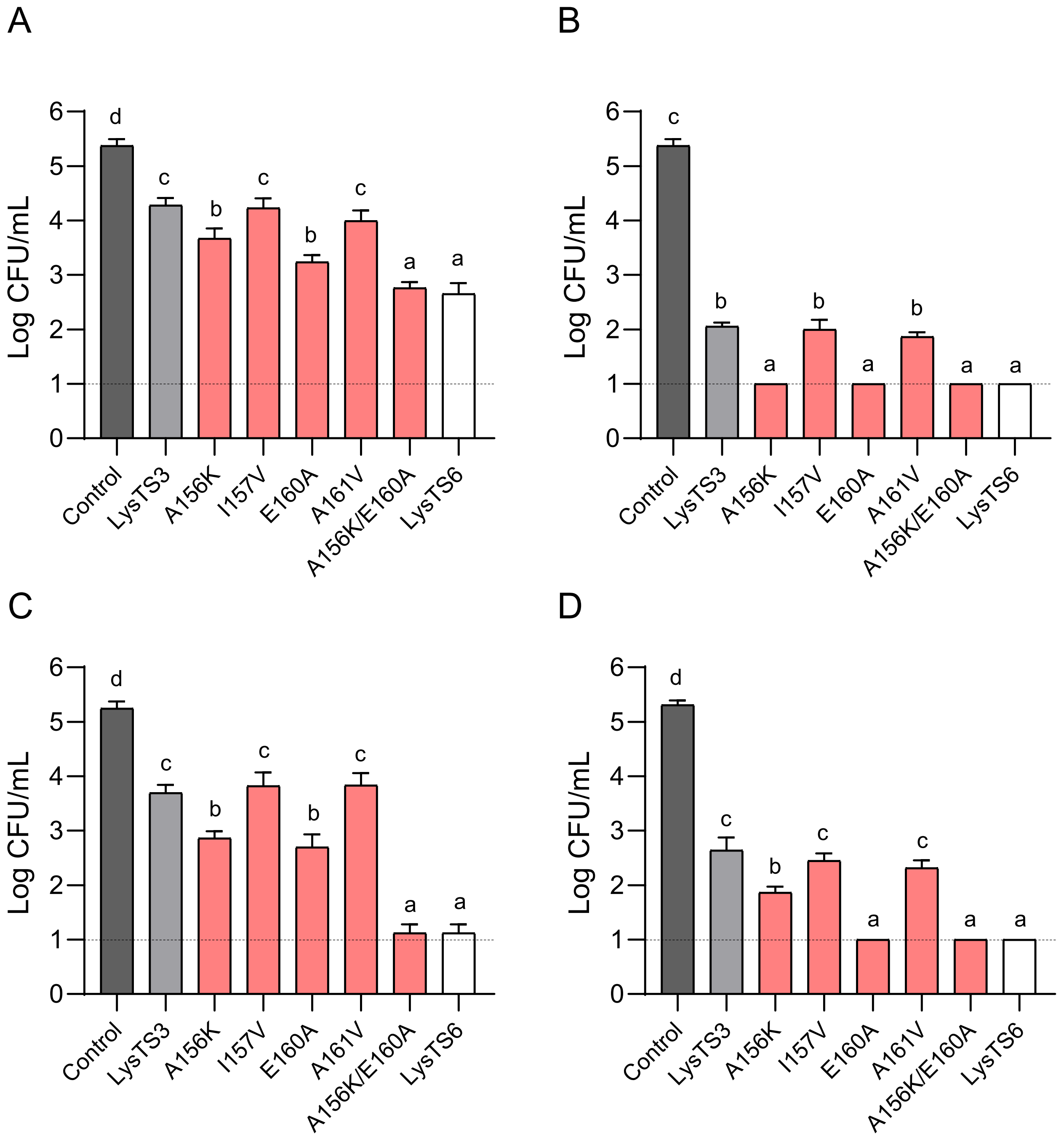

Figure S7. Antibacterial activity of LysTS3, LysTS6, and LysTS3 mutants. (A) Antibacterial activity of 0.5 μM of LysTS3, LysTS6, and LysTS3 mutants with various amino acid residue substitutions against *E. coli* after 1 h of treatment. (B) Antibacterial activity of 1 μM of LysTS3, LysTS6, and LysTS3 mutants with various amino acid residue substitutions against *E. coli* after 1 h of treatment. (C) Antibacterial activity of 1 μM of LysTS3, LysTS6, and LysTS3 mutants with various amino acid residue substitutions against *E. coli* after 15 min of treatment. (D) Antibacterial activity of 1 μM of LysTS3, LysTS6, and LysTS3 mutants with various amino acid residue substitutions against *E. coli* after 30 min of treatment. All experiments were repeated in triplicate and the dotted lines indicate the limit of detection. Statistical significance was analyzed by one-way analysis of variance (ANOVA) with Tukey's multiple-comparison test among the experimental groups. The different letters indicate statistically significant differences.

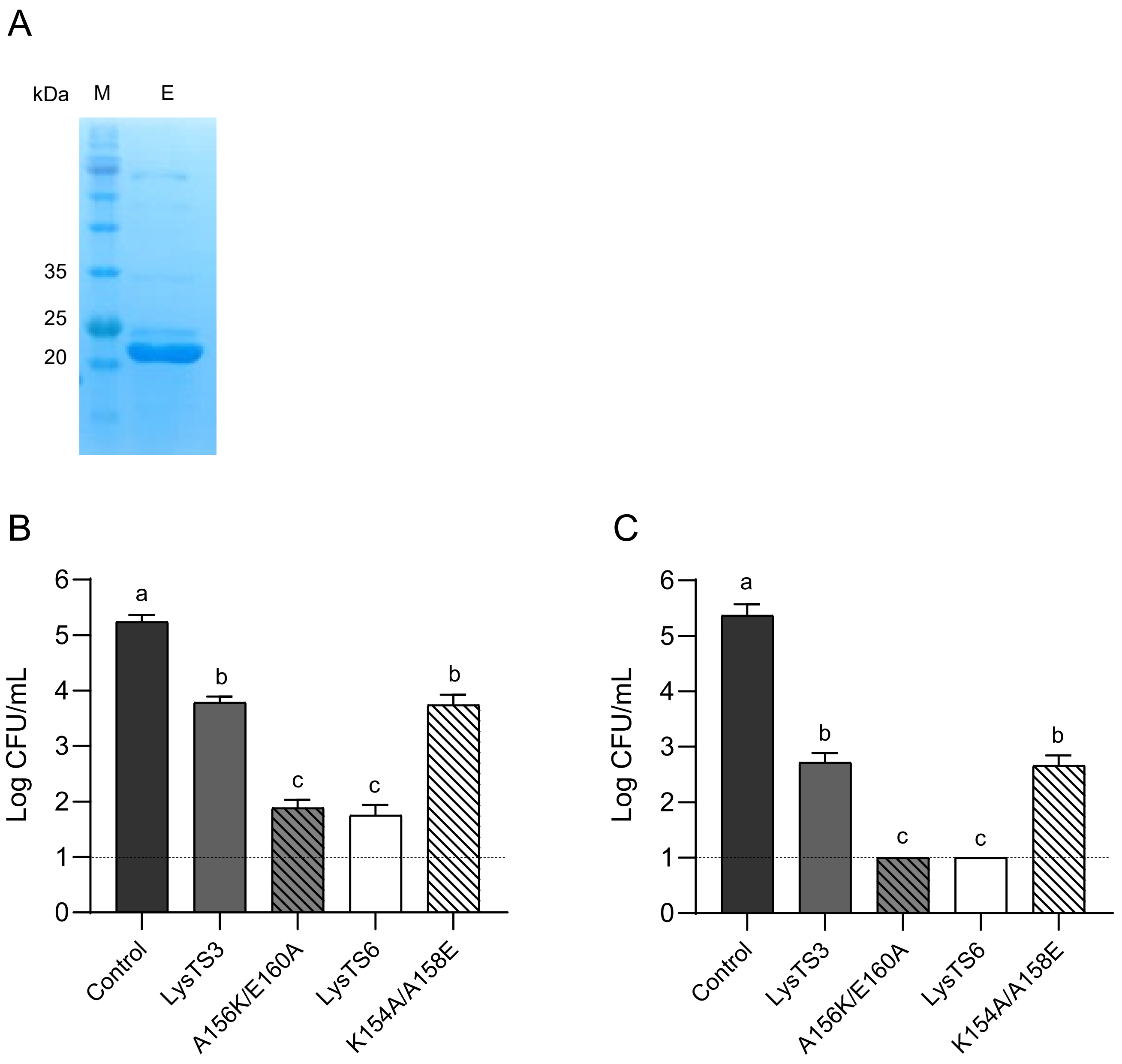

Figure S8. SDS-PAGE analysis of K154A/A158E and antibacterial activity of LysTS3, A156K/E160A, LysTS6, and K154A/A158E. (A) SDS-PAGE analysis of K154A/A158E. The purified K154A/A158E was analyzed on 15% SDS-PAGE gel. M, molecular weight marker; E, K154A/A158E. (B) Antibacterial activity of 1 μM of LysTS3, A156K/E160A, LysTS6 and K154A/A158E against *E. coli* after 15 min of treatment. Exponential phase *E. coli* were treated with 1 μM of each endolysin and incubated at 37°C for 15 min. (C) Antibacterial activity of 1 μM of LysTS3, A156K/E160A, LysTS6 and K154A/A158E against *E. coli* after 30 min of treatment. Exponential phase *E. coli* were treated with 1 μM of each endolysin and incubated at 37°C for 30 min. All experiments were repeated in triplicate and the dotted lines indicate the limit of detection. Statistical significance was analyzed by one-way analysis of variance (ANOVA) with Tukey's multiple-comparison test among the experimental groups. The different letters indicate statistically significant differences.

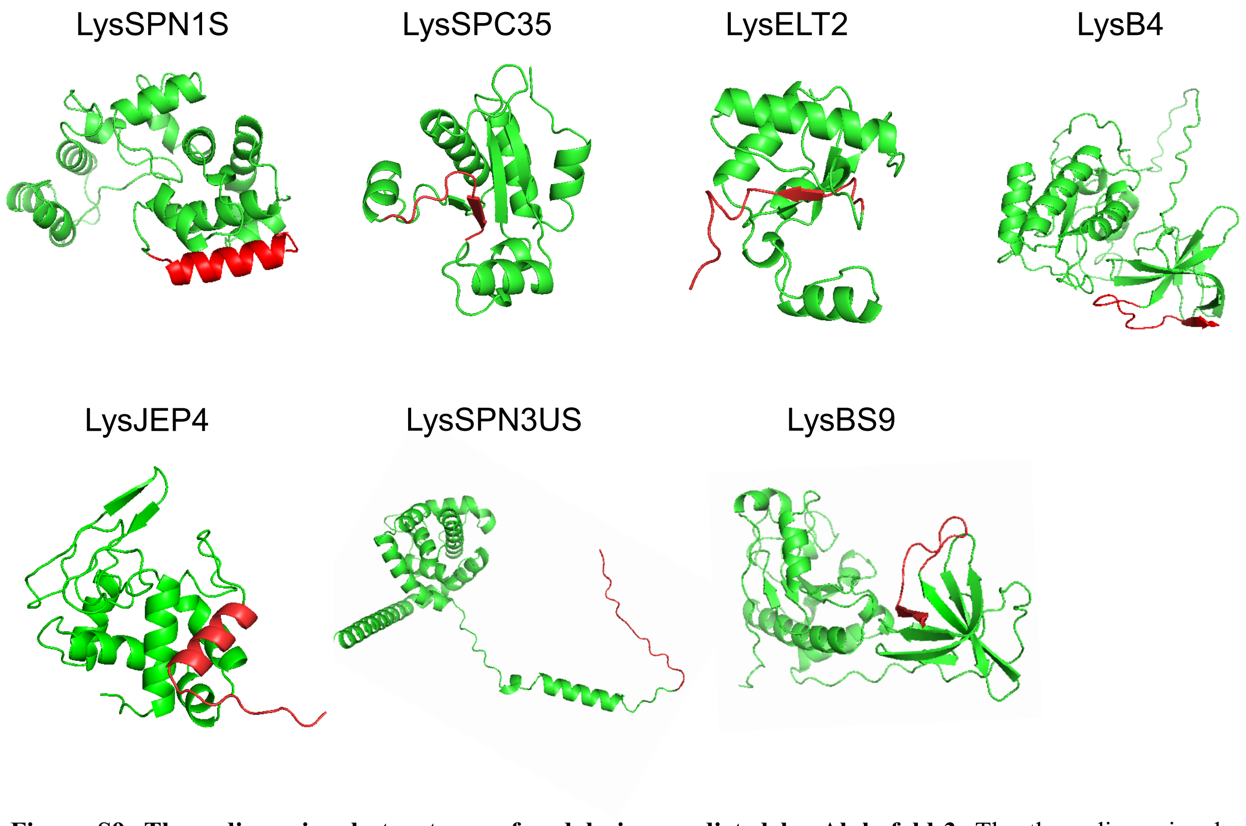

Figure S9. Three-dimensional structures of endolysins predicted by Alphafold-3. The three-dimensional structures of endolysins available in our laboratory (LysSPN1S, LysSPC35, LysELT2, LysB4, LysJEP4, LysSPN3US, and LysBS9) were predicted by Alphafold-3. The amino acid residues at the C-terminus are shown in red. LysSPN1S and LysJEP4 have clear helical structures at the C-terminus.

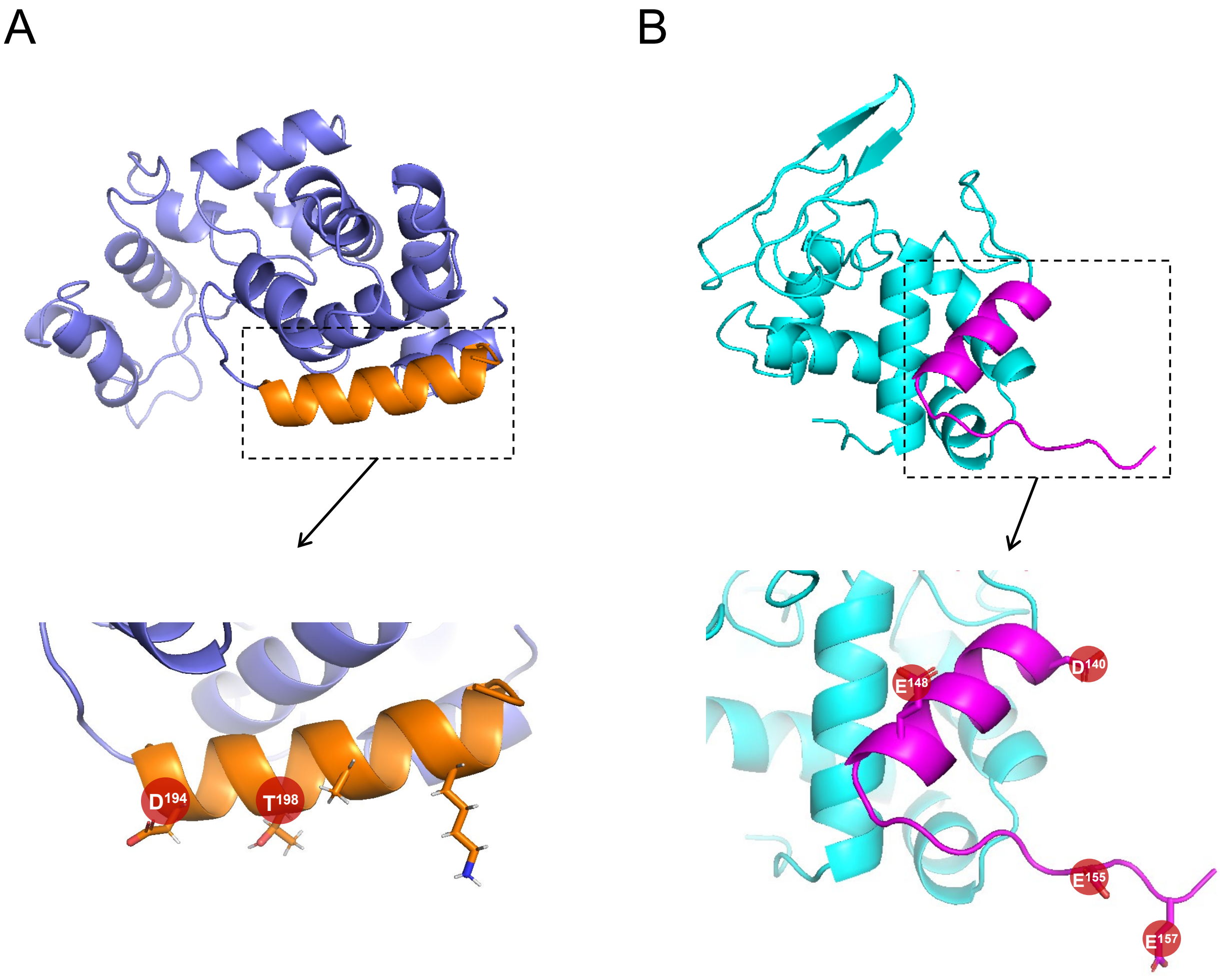

Figure S10. Detailed three-dimensional structures of the C-terminal amphipathic helix of LysSPN1S and LysJEP4 predicted by Alphafold-3. (A) The three-dimensional structures of the C-terminal amphipathic helix (amino acid residues from 191 to 207) of LysSPN1S predicted by Alphafold-3. The C-terminal helix of LysSPN1S is shown in orange. The locations of the surface-exposed negatively charged amino acid residue Asp_{194} and uncharged amino acid residue Thr_{198} on the C-terminal amphipathic helix of LysSPN1S are shown as red circles. (B) The three-dimensional structures of C-terminal amphipathic helix (amino acid residues from 139 to 158) of LysJEP4 predicted by Alphafold-3. The C-terminal helix of LysJEP4 is shown in pink. The locations of the surface-exposed negatively charged amino acid residues Asp_{140}, Glu_{148}, Glu_{155}, and Glu_{157} on the C-terminal amphipathic helix of LysJEP4 are shown as red circles.

A

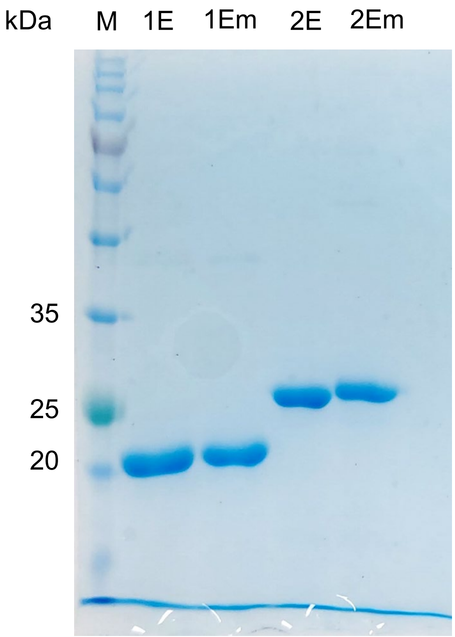

B

Endolysin	Muralytic activity (Unit/mg)
LysSPN1S	5,664
LysSPN1Sm	5,422
LysJEP4	23,142
LysJEP4m	18,544

Figure S11. SDS-PAGE analysis of LysSPN1S, LysSPN1Sm, LysJEP4, LysJEP4m and their muralytic activities. (A) SDS-PAGE analysis of LysSPN1S, LysSPN1Sm, LysJEP4, and LysJEP4m. Purified endolysins were analyzed on 12% SDS-PAGE gel. M, molecular weight marker; 1E, LysJEP4; 1Em, LysJEP4m; 2E, LysSPN1S; 2Em, LysSPN1Sm. (B) The muralytic activity of LysSPN1S, LysSPN1Sm, LysJEP4, and LysJEP4m.

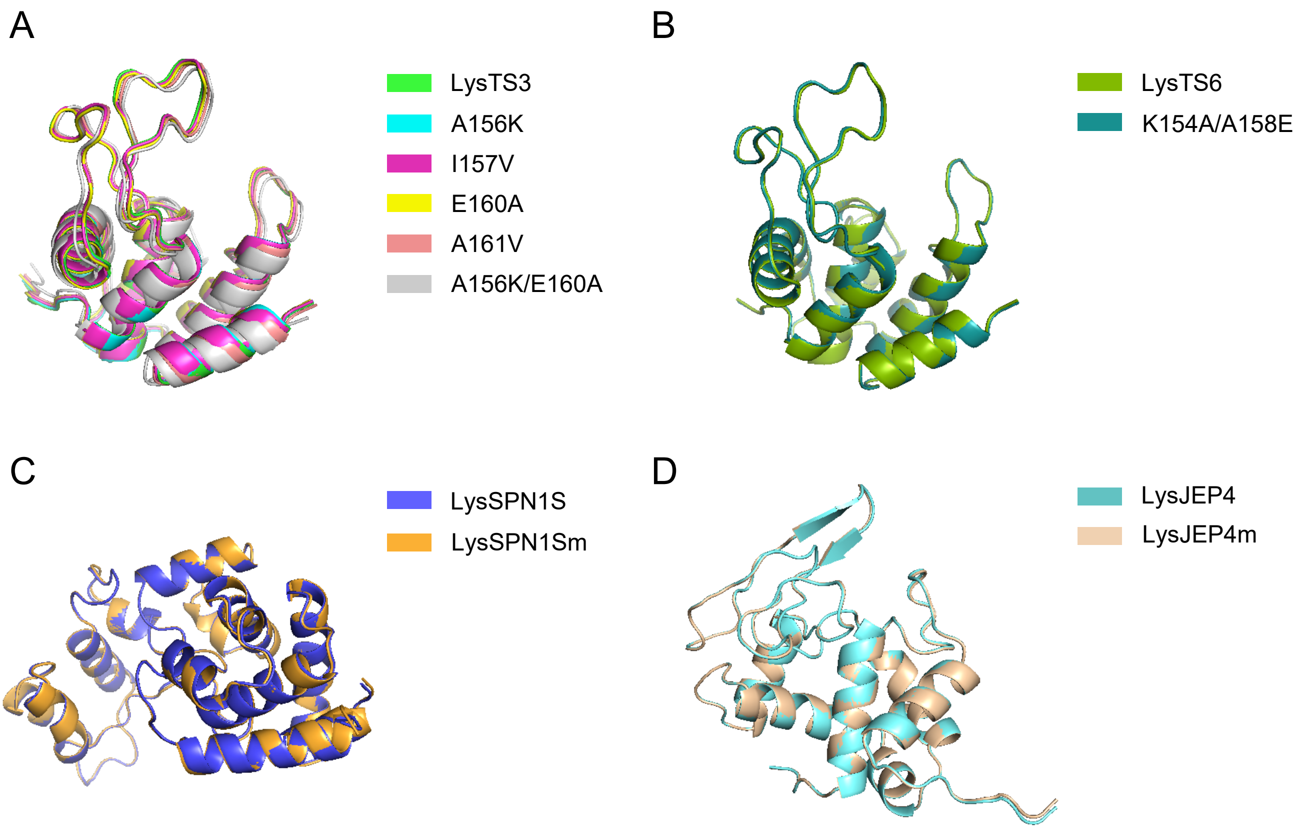

Figure S12. Structural comparison of wild-type endolysins and their mutants. The three-dimensional structures of LysTS3, LysTS6, LysSPN1S, LysJEP4, and their mutants were predicted by Alphafold-3 and aligned. (A) Structural alignment of LysTS3 with its mutants A156K, I157V, E160A, A161V, and A156K/E160A. (B) Structural alignment of LysTS6 with its mutant K154A/A158E. (C) Structural alignment of LysSPN1S with its mutant LysSPN1Sm. (D) Structural alignment of LysJEP4 with and its mutant LysJEP4m.

Table S1. Bacterial strains used in this study

Strain[a]	Antibiotic resistance[b]	Sources
**- *E. coli* strains**		
Escherichia coli ATCC 25922	Unknown	ATCC
Escherichia coli NCCP 13721	AMP	NCCP
Escherichia coli NCCP 15739	AMP	NCCP
Escherichia coli ATCC 43895	Unknown	ATCC
Escherichia coli NCCP 15647	AMP, CIP, GEN, NAL, SXT, TET	NCCP
Escherichia coli NCCP 14038	Unknown	NCCP
Escherichia coli NCCP 14039	AMP, SXT, TET, FOX	NCCP
Escherichia coli clinical isolate no. 11	AN, ATM, CAZ, CIP, FEP, GEN, IPM, MEM, PIP, TZP	Stool
Escherichia coli clinical isolate no. 16	ATM, CAZ, CIP, FEP, IPM, MEM, PIP, TZP	Sputum
Escherichia coli O157:H7 ATCC 35150	Unknown	ATCC
**- Other Gram-negative bacteria**		
Salmonella Typhimurium UK-1	Unknown	ATCC
Salmonella Typhimurium SL1344	Unknown	ATCC
Salmonella Enteritidis ATCC 13076	Unknown	ATCC
Klebsiella pneumoniae KCTC 2242	Unknown	KCTC
Acinetobacter baumannii NCCP 15989	CC, OXA, ST, MEM, DOX, GEN, ERY	NCCP
Acinetobacter baumannii NMS 1915	CIP, COL, MEM	NCCP
Acinetobacter baumannii NCCP 15991	CC, OXA, ST	NCCP
Pseudomonas aeruginosa NCCP 17542	AN, ATM, CAZ, CIP, COL, DOR, FDC, FEP, GEN, I-R, IPM, L VX, MEM, TOB, TZP	NCCP
Cronobacter sakazakii ATCC 29544	Unknown	ATCC

[a]ATCC, American Type Culture Collection; KCTC, Korean Collection for Type Cultures; NCCP, National Culture Collection for Pathogens.

[b]AMC, Amoxicillin; AMP, Ampicillin; AN, Amikacin; ATM, Aztreonam; CAZ, Ceftazidime; CC, Clindamycin; CIP, Ciprofloxacin; COL, Colistin; DOR, Doripenem; DOX, Doxycycline; ERY, Erythromycin; FDC, Cefiderocol; FEP, Cefepime; FOX, Cefoxitin; GEN, Gentamicin; I-R, Imipenem-relebactam; IPM, Imipenem; MEM, Meropenem; NAL, Nalidixic acid; OXA, Oxacillin; PIP, Piperacillin; ST, Streptomycin; SXT, Trimethoprim; TET, Tetracycline; TOB, Tobramycin; TZP, Piperacillin + Tazobactam.

Table S2. Plasmids used in this study

Plasmids	Characteristics
pET28a::LysTS3	Kan^R, T7 Promoter, IPTG induced, N-terminal hexa his-tagged, endolysin LysTS3
pET28a::LysTS3Δα2-LysTS6α2	Kan^R, T7 Promoter, IPTG induced, N-terminal hexa his-tagged, endolysin LysTS3, C-terminal α2 helix of LysTS3 changed to C-terminal α2 helix of LysTS6
pET28a::LysTS3Δ(α1-α2)-LysTS6(α1-α2)	Kan^R, T7 Promoter, IPTG induced, N-terminal hexa his-tagged, endolysin LysTS3, C-terminal α1-α2 helices of LysTS3 changed to C-terminal α1-α2 helices of LysTS6
pET28a::LysTS3ΔCTD-LysTS6CTD	Kan^R, T7 Promoter, IPTG induced, N-terminal hexa his-tagged, endolysin LysTS3, CTD of LysTS3 changed to CTD of LysTS6
pET28a::LysTS6	Kan^R, T7 Promoter, IPTG induced, N-terminal hexa his-tagged, endolysin LysTS6
pET28a::A156K	Kan^R, T7 Promoter, IPTG induced, N-terminal hexa his-tagged, endolysin LysTS3, 156th Alanine (A) of LysTS3 changed to Lysine (K)
pET28a::I157V	Kan^R, T7 Promoter, IPTG induced, N-terminal hexa his-tagged, endolysin LysTS3, 157th Isoleucine (I) of LysTS3 changed to Valine (V)
pET28a::E160A	Kan^R, T7 Promoter, IPTG induced, N-terminal hexa his-tagged, endolysin LysTS3, 160th Glutamic acid (E) of LysTS3 changed to Alanine (A)
pET28a::A161V	Kan^R, T7 Promoter, IPTG induced, N-terminal hexa his-tagged, endolysin LysTS3, 161th Alanine (A) of LysTS3 changed to Valine (V)
pET28a::A156K/E160A	Kan^R, T7 Promoter, IPTG induced, N-terminal hexa his-tagged, endolysin LysTS3, 156th Alanine (A) and 160th Glutamic acid (E) of LysTS3 changed to Lysine (K) and Alanine (A), respectively
pET28a::K154A/A158E	Kan^R, T7 Promoter, IPTG induced, N-terminal hexa his-tagged, endolysin LysTS6, 154th Lysine (K) and 158th Alanine (A) of LysTS6 changed to Alanine (A) and Glutamic acid (E), respectively
pET28a::LysSPN1S	Kan^R, T7 Promoter, IPTG induced, N-terminal hexa his-tagged, endolysin LysSPN1S
pET28a::LysSPN1Sm	Kan^R, T7 Promoter, IPTG induced, N-terminal hexa his-tagged, endolysin LysSPN1S, 194th Aspartic acid (D), 198th Threonine (T) of LysSPN1S changed to Lysine (K)
pET28a::LysJEP4	Kan^R, T7 Promoter, IPTG induced, N-terminal hexa his-tagged, endolysin LysJEP4
pET28a::LysJEP4m	Kan^R, T7 Promoter, IPTG induced, N-terminal hexa his-tagged, endolysin LysJEP4, 140th Aspartic acid (D), 148th, 155th, and 157th Glutamic acid (E) of LysJEP4 changed to Lysine (K)

Table S3. Muralytic activities of LysTS3, LysTS6 and their amino acid mutants

Endolysin	Muralytic activity (Unit/mg)
LysTS3	17,050
A156K	16,138
E160A	16,500
A156K/E160A	15,951
I157V	15,893
A161V	15,092
LysTS6	17,108
K154A/A158E	15,756
